# Thermal Study and Emission Characteristics of Rice Husk Using TG-MS

**DOI:** 10.3390/ma14206203

**Published:** 2021-10-19

**Authors:** José Ignacio Arranz, María Teresa Miranda, Irene Montero, Francisco José Sepúlveda

**Affiliations:** Department of Mechanical, Energy and Materials Engineering, School of Industrial Engineering, University of Extremadura, Av. Elvas, s/n, 06006 Badajoz, Spain; tmiranda@unex.es (M.T.M.); imontero@unex.es (I.M.); fsepulveda@unex.es (F.J.S.)

**Keywords:** rice husk, thermogravimetry, spectrometry mass, emission, kinetic analysis, biomass

## Abstract

Rice husks are a by-product that is generated in large quantities in Spain. However, they are not used efficiently. One of their possible applications is its thermal use in power generation equipment. For that purpose, it is important to know the characteristics of rice husks and their thermal behavior, as well as their possible pollutant emission to the atmosphere with respect to its thermal use as a biofuel. In this work, the thermal characteristics of rice husks and their thermal behavior were studied by using thermogravimetry and mass spectroscopy for two different atmospheres (oxidizing and inert). This way, the thermal profiles and the main characteristics were studied, as well as the emission of possible pollutants to the atmosphere, such as CO_2_, CH_4_, NO_2_, NH_3_, SO_2_, and H_2_S. Moreover, three different methods (FWO, KAS, and Starink) were used to carry out a thermal analysis, in order to obtain the main thermal parameters such as activation energy. The results of the analysis predicted that rice husks could be used as biofuel in industrial thermal equipment based on its acceptable calorific value, good thermal characteristics, and low gas emissions both in oxidizing and inert atmosphere (although they have a high ash content).

## 1. Introduction

Spain is a country with a high agricultural production, large cultivation areas, and a considerable number of agro-food industries, wherein large amounts of waste are generated due to the normal development of their activities. Recently, a significant advance has taken place in the management of biomass waste. In fact, terms such as by-product (replacing waste) are usually used, thanks to the implementation of steps that allow a suitable recycling of wastes [1]. However, there are no global solutions that might be applicable to all these by-products, with some solutions causing environmental problems if they are not suitably managed.

Every year, large amounts of agricultural wastes are not properly used in Spain. In particular, the rice industry has produced over 789,000 t of this product in 2019 in this country [2]. The main waste that is generated is the husk that covers the grains of the rice. This by-product does not have an application in the elaboration of concentrated feed for animals due to its high silica content (SiO_2_) [3], which significantly reduces its digestibility [4]. Due to its physical-chemical constitution, rice husks also represent a waste of very difficult biodegradation. This fact, apart from the fact that in rice processing plants the amount of husk generated is around 20 wt.% of total production, and considering the very low specific weight of the bulk husk (100 kg m^−3^), results in the evacuation and the transport of rice husks representing a major problem that implies high costs and a harmful impact to the environment [5].

In order to solve these problems and promote the use of clean and efficient energy from agricultural waste in rural areas, the use of this kind of waste (such as rice husks) as an energy source through thermal processes can be an interesting solution. The use of this by-product as a source of energy generation represents an important advance in this direction, viable from a technical point of view, as well as convenient from an economic and ecological point of view [6].

Some authors have studied the thermal characterization of rice husks and their behavior during combustion through several techniques [7,8,9]. On the other hand, other thermal processes, such as the pyrolysis of this by-product, have been studied by other researchers [3,10,11]. Gasification processes as a method for thermal use of rice husks was also investigated [12,13,14]. However, there are no studies simultaneously dealing with energy and environmental aspects of this by-product, analyzing its behavior both in oxidizing and inert atmosphere.

The simultaneous use of thermogravimetric analysis and mass spectroscopy has attracted the interest of researchers due to its versatility and quickness, providing semi-quantitative information [15].

On the other hand, in order to design energy conversion systems for biomass, it is necessary the use of kinetic studies [16]. This way, some kinetic methods, such as Flynn-Wall-Ozawa (FWO) [17], Kissinger-Akahira-Sunose (KAS) [18,19] or Starink method [20], are popular due to their ease to obtain some parameters and therefore to understand the mechanisms of thermal processes [21,22,23,24,25]. However, most kinetic studies are focused on pyrolysis of this kind of products.

In this work, the possibilities offered by rice husks as a raw material for their use as a biofuel were studied. This study was carried out through two ways: determining the thermal profiles of the by-product through thermogravimetry, followed by the kinetic analysis through FWO, KAS, and Starink methods; and determining the gas emissions that could be problematic for the environment by using mass spectrometry in two different atmospheres (oxidizing and inert). This way, it would be possible to obtain a wide knowledge about thermal properties and environmental aspects, establishing the foundations for a possible thermal use of this by-product.

## 2. Materials and Methods

### 2.1. Sample Preparation

To carry out this study, rice husks were obtained from a rice company in the region of Extremadura (Miajadas, Spain). It was not necessary to carry out any drying or conditioning pre-treatment, since the by-product has a moisture level compatible with the analysis (<10 wt.%). Only grinding was carried out to reduce the particle size of the samples to less than 250 μm for characterization, thermogravimetry, and mass spectrometry tests.

### 2.2. Sample Characterization

To obtain the properties of the rice husk samples, different characterization tests (shown in Table 1) were carried out, according to the indicated standards.

### 2.3. TG-MS Analysis

In this study, the thermogravimetric experiments were carried out by using a thermobalance with a maximum sensibility of 5 μg and an accuracy higher than 0.2% of total weight and temperature (SDT Q600, TA Instruments, New Castle, DE, USA). All of the tests were carried out in inert atmosphere (argon) and oxidizing atmosphere (synthetic air) at 100 mL min^−1^ and for five linear heating rates (5, 10, 20, 30, 40 °C min^−1^). The weight used for the experiments was 10.0 ± 0.1 mg, and the particle size was below 250 μm, in order to assure that the experiments would be carried out in the kinetic regime, eliminating heat and mass transfer effects on the results [33,34]. The analysis was made in a temperature range of 20–800 °C.

Thermogravimetric-mass spectrometric (TG/MS) analysis was also carried out in order to analyze qualitatively the evolved volatiles during combustion and pyrolysis of the examined samples. This study was carried out by using a coupled TG-MS system (Thermogravimetric system, SDT Q600, TA Instruments, New Castle, DE, USA; Mass Spectrometer, Pfeiffer Tecnovac Thermostar GDS301 T3, Tecnovac, Madrid, Spain). A fused silicon capillary line was used as the pressure reducing system and for the transfer of the evolved volatiles of pyrolysis to the MS. The capillary was continuously heated at 200 °C to prevent the condensation of the gaseous products in it. The mass to charge ratios were the following: m/z = 16, 44 (attributed to carbon-based evolved gases, CH_4_ and CO_2_, respectively); 17, 46 (attributed to nitrogen-based gases, NH_3_ and NO_2_, respectively); and 34, 64 (attributed to sulphur-based evolved gases, H_2_S and SO_2_, respectively). These ratios were continuously monitored.

### 2.4. Kinetic Analysis

Various thermal characteristics, such as activation energy, were calculated using three different methods (FWO, KAS and Starink). Concerning the kinetic analysis, the FWO method was used, in order to check the mechanism that was selected [17]. The kinetics of pyrolysis of biomass can be expressed by a single-step reaction [16]. This kinetic model can be described by the following equation (Equation (1)):

(1) $$\frac{d\alpha }{dt}=Aexp\left(\frac{-E_{a}}{RT}\right)f\left(\alpha \right)$$

where *dα*/*dt* is the rate of reaction; *A*, *E_a_*, and *R* are the pre-exponential factor (min^−1^), activation energy (kJ mol^−1^), and universal gas constant (8.314 J (K mol)^−1^), respectively; and *f*(*α*) is the conversion function used by the reaction model, depending on the control reaction mechanism and *α* is the conversion degree.

For non-isothermal conditions in a linear heating rate, the FWO assumes that the activation energy is constant for a certain duration of the reaction, obtaining Equation (2):

(2) $$g\left(\alpha \right)=\int_{0}^{\alpha }\frac{d\alpha }{f\left(\alpha \right)}=\frac{A}{\Phi }\int_{0}^{T_{\alpha }}exp\left(\frac{-E_{a}}{RT}\right)dT$$

The integral of temperature does not have an exact analytical solution [35]. Nevertheless, it can be approximated by the empirical interpolation proposed by Doyle [36] and applying the natural logarithm to Doyle’s approximation the following equation is obtained:

(3) $$\ln\left(\beta_{i}\right)=\ln\left(\frac{A_{\alpha }E_{\alpha }}{Rg\left(\alpha \right)}\right)-5.331-1.052\frac{E_{\alpha }}{RT_{\alpha i}}$$

where *g*(*α*) is a constant for each conversion value. The sub-indexes *i* and *α* indicate different values of heating rate (*β*) and degree of conversion (α), respectively. The activation energies were calculated from the slope −1.052E_α_/*R* generated when *ln*(*β_i_*), natural logarithm of heating rates versus 1000/*T_αi_* was plotted.

On the other hand, the method developed by KAS [18,19] is described as follows:

(4) $$\ln\left(\frac{\beta_{i}}{T^{2}}\right)=\ln\left(\frac{A_{\alpha }R}{E_{\alpha }g\left(\alpha \right)}\right)-\frac{E_{\alpha }}{RT_{\alpha i}}$$

This way, the activation energy can be calculated by representing *ln*(*β*/*T*^2^) versus 1000/*T*.

Starink [20] analyzed FWO and KAS methods jointly and concluded that both methods can be adjusted to Equation (5):

(5) $$\ln\left(\frac{\beta_{i}}{T^{s}}\right)=C_{s}-\frac{BE_{\alpha }}{RT_{\alpha i}}$$

where, for FWO method, *s* = 0 and *B* = 0.457 and, for KAS method, *s* = 2 and *B* = 1. By optimizing constants, Starink proposed the following values: *s* = 1.0037 and *B* = 1.8. Therefore, the Starink method can be expressed as follows:

(6) $$\ln\left(\frac{\beta_{i}}{T^{1.8}}\right)=C_{s}-1.0037\frac{E_{\alpha }}{RT_{\alpha i}}$$

Consequently, the activation energy can also be calculated by plotting ln(*β*/*T*^1.8^) versus 1000/*T*.

## 3. Results and Discussion

### 3.1. Characterization of the By-Product

Table 2 shows the characterization results for the rice husks.

It should be pointed out the low percentages of N and S in rice husks in the ultimate analysis, which will imply low levels of the corresponding nitrogen and sulphur-based emissions. Chen et al. [37] showed an N value almost equal (0.48%) to that obtained in this work. Authors like Wang et al. [38] reported similar values for N and S, although slightly higher (0.69 % and 0.01 %, respectively). Other positive aspect was the heating value, exceeding 18 MJ kg^−1^, very similar value compared to those reported by Lopes Grotto et al. [39] for rice husk from two types of genetically improved rice. Other authors, such as Su et al. [3] reported an HHV value of slightly more than 15 kJ kg^−1^.

The negative note is the high ash content obtained in the proximate analysis. This circumstance should be taken into account when using this by-product in a thermal generation equipment. Nevertheless, other authors, like Worasuwannarak et al. [40] and Chen et al. [41] showed ash contents above 13 %. Moreover, its bulk density is too low, and therefore the long-distance transportation of this by-product would not be economically efficient.

### 3.2. Thermal Decomposition

**Figure 1:**
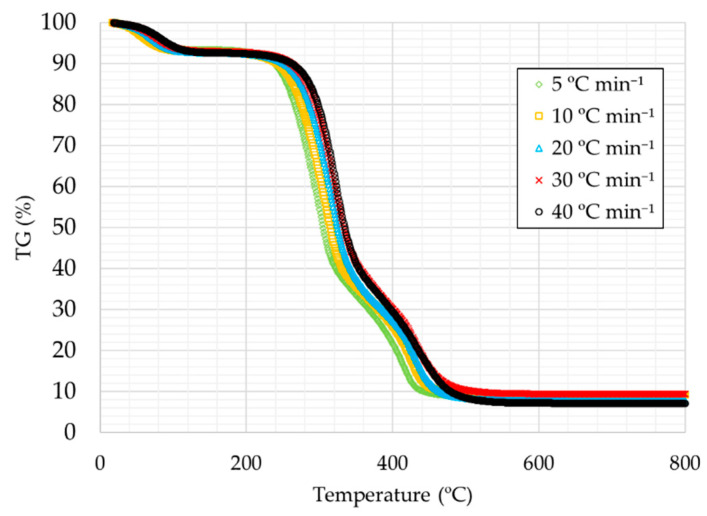
TG curves for the heating rates studied under oxidative atmosphere.

**Figure 2:**
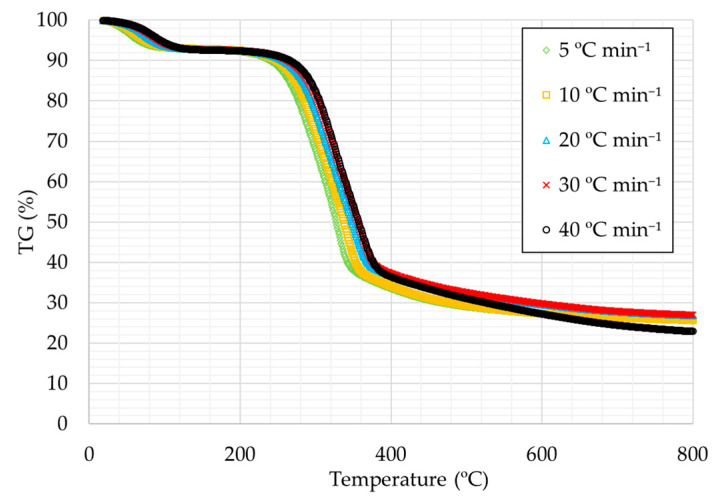
TG curves for the heating rates studied under inert atmosphere.

Figure 1 and Figure 2 present the thermogravimetry curves (TG) for rice husks for all the heating rates studied, under oxidative atmosphere (Figure 1) and under inert atmosphere (Figure 2), respectively.

The curves are very similar in all cases, with the first decrease being caused by the removal of moisture, followed by a very pronounced decrease due to the removal of volatiles in the first instance and finally to the degradation of fixed carbon. The curves show at higher temperatures that there is a certain percentage that is not degraded, which corresponds to the by-product ash in the case of Figure 1 and to the addition of ash and fixed carbon in the case of Figure 2.

It can also be observed that as the heating ramp was higher the thermal decomposition temperatures are slightly higher, a trend that can be observed in TG curves of many substances [42].

**Figure 3:**
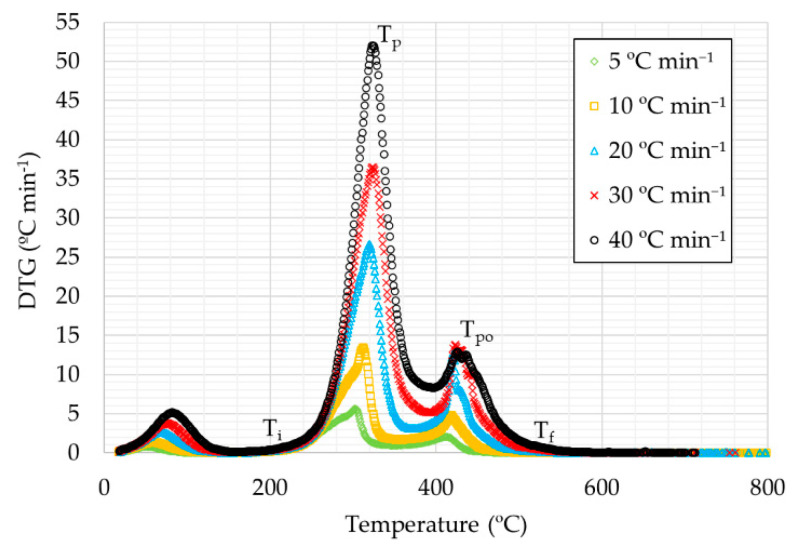
DTG curves for the heating rates studied under oxidative atmosphere.

**Figure 4:**
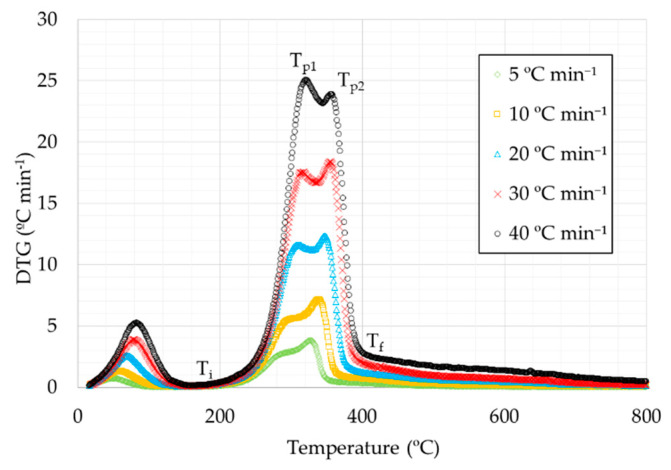
DTG curves for the heating rates studied under inert atmosphere.

Derivative thermogravimetry curves (DTG) for rice husks are shown in Figure 3 and Figure 4. It can be seen that the curves for the different heating rates were similar, consisting in a small peak in the initial part, a more pronounced peak in the intermediate temperature range, and a long final peak. In oxidizing atmosphere, a third peak can also be observed.

Based on the whole temperature range considered, samples could be separated into three different stages, according to each peak in DTG curve. The first stage corresponds to the lowest temperature range, from room temperature up to 177–217 °C, depending on the heating rate. In this stage a small weight loss is produced, due to the water loss on account of evaporation and the release of some light volatiles [43]. The second step corresponds to the highest weight loss, caused by devolatilization, and produced in the temperature range between 177–217 and 372–408 °C. The plots showed a double peak in the case of inert atmosphere, where the highest peak corresponds to the highest temperature. This fact was not observed for the plot at the highest heating rate (40 °C min^−1^), as the highest peak took place at lower temperature. Moreover, in the two first curves (5 and 10 °C min^−1^, respectively), the temperature of the first peak can not be established. The third stage is related to the highest temperatures from 372–408 °C. This stage is the most different one between oxidative and inert atmosphere. Regarding oxidizing atmosphere curves, a peak was observed, corresponding to the weight loss due to char combustion. The final waste is related to the ash amount of the sample. In the curves corresponding to inert atmosphere, the weight loss is too slow, possibly due to the decomposition of carbonaceous materials contained in the by-product [44].

Considering the chemical composition of rice husks, the second step is usually attributed to the degradation of hemicellulose and cellulose and the third step to the degradation of lignin. Some authors show a chemical composition of rice husks consisting of 20–21% hemicellulose, 29–38% cellulose, and 15–20% lignin [37,45].

The main characteristics for thermal decomposition are shown in Table 3:

It can be observed that, in all cases, as the heating rate increased, the peaks were obtained at higher temperatures except for the heating rate at 40 °C min^−1^ in inert atmosphere, where this effect was observed in the first peak and not in the second one as in the remaining cases. Moreover, it can be observed in the corresponding figure at inert atmosphere that the first peak of the second stage was more and more pronounced, with the highest peak for the curve corresponding to the highest heating rate. Moreover, DTG_max_ values were higher for higher heating rates, for both atmospheres, obtaining higher values for the tests in oxidizing atmosphere.

### 3.3. Kinetic Analysis

**Figure 5:**
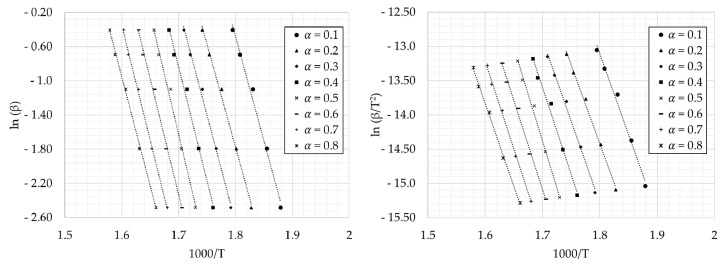

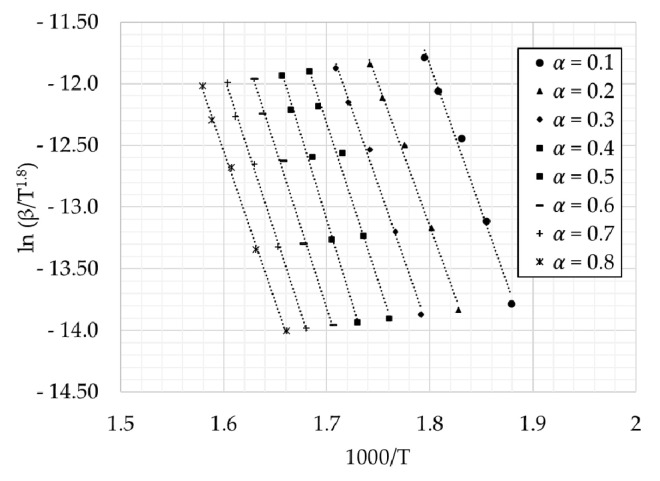
FWO, KAS, and Starink plots for the values of conversion degree.

For a correct use of the model, the appropriate temperature ranges are the ones between T_i_ and T_f_ (see Table 3), corresponding to the tests in inert atmosphere. Conversion values (*α*) were chosen in the same range (0.1–0.8) for all the heating rates. FWO, KAS, and Starink plots are shown in Figure 5.

By using the values obtained from Figure 5, the subsequent equations corresponding to the model used can be obtained. Therefore, the activation energies from the slope of these lines are calculated. The values of activation energy and their corresponding correlation coefficients, obtained in this research work, are shown in Table 4.

The activation energy is the minimum value of energy that is necessary to start the reaction. Therefore, a reaction will be more difficult to be started when the activation energy is higher. The activation energy calculated by FWO method was in a range of 193.87–223.67 kJ moL^−1^, with an average value of 205.64 kJ moL^−1^. For KAS method, *E_a_* was between 194.90 and 225.26 kJ moL^−1^, with an average value of 206.61 kJ moL^−1^. Finally, *E_a_* values obtained by using the Starink method were very similar to the former (between 195.08 and 225.63 kJ moL^−1^, and an average value of 206.82 kJ moL^−1^). In turn, a slight increase in *E_a_* as α was higher, decreasing slightly from *α* = 0.5. This trend was repeated in all the methods used. In general, an increase in the activation energy with temperature is due to parallel and complex reactions [46,47].

In almost all cases, the correlation coefficients exceeded the value of 0.990, which were considered acceptable, being higher for the highest degrees of conversion. Nevertheless, these values were lower for intermediate conversions. This irregular trend is, in general, due to the abovementioned complex reactions.

**Figure 6:**
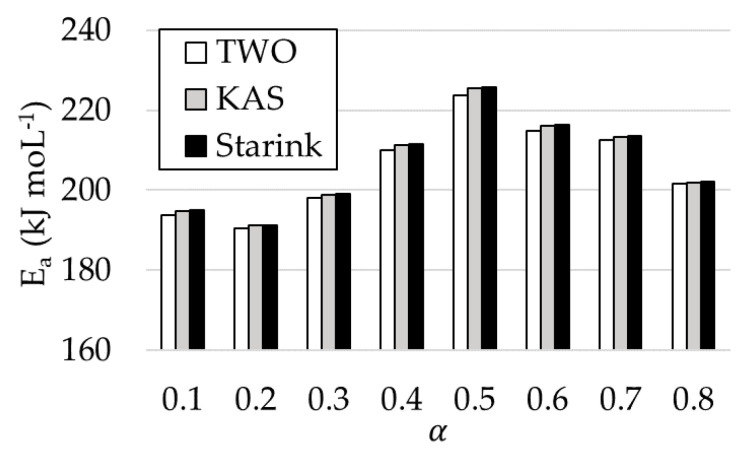
Activation energy obtained by FWO, KAS, and Starink methods, at different conversion rates.

Figure 6 shows the activation energy values obtained for the three methods used at different conversion degrees. The patterns were similar, to the values obtained by the Starink method being higher than the ones obtained by using the remaining methods.

Gai et al. [48] obtained *E_a_* values for rice husks at different conversion degrees through the Starink method. These values were between 88.99 and 50.49 kJ moL^−1^, with an average value of 79 kJ moL^−1^. All these values were substantially below the values obtained in this work. Gai also calculated the activation energy for corn stover by the Starink method, obtaining a value of 129 kJ moL^−1^. On the contrary, Jia et al. [49] obtained very similar results through the FWO method for the by-product studied in this study, with a maximum value of 219.80 kJ moL^−1^ and a minimum of 194.37 kJ moL^−1^, for a conversion degree (*α*) range between 0.1 and 0.8.

Özsin et al. [46] obtained activation energy values for several by-products obtained through the same methods studied in this research work. For chestnut shells, according to the FWO method, a value of 175.9 kJ moL^−1^ was obtained; according to the KAS method, 175.2 kJ moL^−1^, and according to Starink method, 175.5 kJ moL^−1^. These values were below the ones obtained for rice husks, but they were very close. Özsin also obtained activation energy values for cherry stones (*E_a_*: 268.5 kJ moL^−1^ by FWO method, 272.2 kJ moL^−1^ by KAS and Starink method) and for grape seeds (*E_a_*: 187.3 kJ mol^−1^ (FWO), 186.6 kJ moL^−1^ (KAS), 186.9 kJ moL^−1^ (Starink)). Moreover, in all cases, as it happened in this work, *E_a_* values increased with the conversion degree. However, this value decreased at the highest *α* values.

The activation energy obtained by the FWO method for olive pomace was reported by Özveren et al. [50], with a value of 300 kJ moL^−1^, approximately. Nevertheless, Brachi et al. [51] obtained an activation energy value of 210 kJ moL^−1^, approximately. Very different values were obtained by Miranda el al. [52] for the activation energy of grape pomace (31.26, 16.92 and 41.98 kJ moL^−1^, for each stage), obtained by using the Criado method. Other similar wastes, such as coffee ground residues, showed activation energies between 220-250 kJ moL^−1^, approximately [53]. Huang et al. [54] obtained the activation energy of spent mushroom substrate by FWO (*E_a_* = 171.49 kJ moL^−1^) and KAS (*E_a_* = 170.18 kJ moL^−1^) methods, and observed the same trend concerning the degree of conversion. Similarly, Khiari et al. [55] obtained a mean *E_a_* value of 223.87 and 231.21 kJ moL^−1^ for FWO and KAS methods, respectively. Finally, for other kind of biofuel like coal, Otero et al. [56] reported a value of 114.4 kJ moL^−1^ by using the FWO method.

### 3.4. Evolved Gases

**Figure 7:**
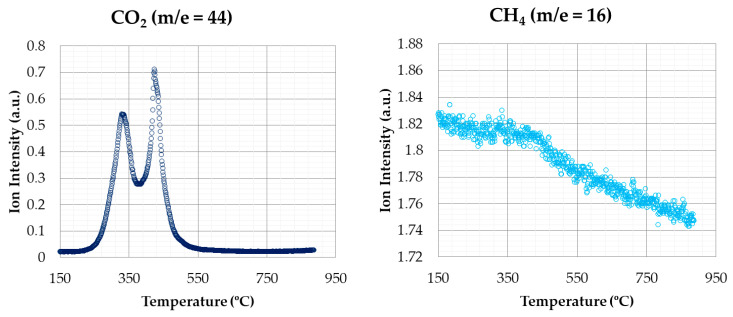
CO_2_ and CH_4_ emissions (oxidizing atmosphere).

The signal variation of the emissions of the gases can be obtained by the sampling capability of the mass spectrometer. Figure 7 shows the emission of CO_2_ and CH_4_ in combustion tests (oxidizing atmosphere) for a heating rate of 20 °C min^−1^.

CO_2_ is one of the most abundant released gases during combustion, and is involved in most of the thermal degradation events. The pattern of CO_2_ shows that the release of this compound was produced both in the second stage (corresponding to devolatilization) and the last one (corresponding to char combustion), with the second stage being higher.

CH_4_ signal hardly showed variations. It can be observed a slight increase around 350–450 °C, approximately. In this case, the different stages are not as defined as in the case of CO_2_. Therefore, CH_4_ emissions, although having a weak signal, are produced during the whole combustion process.

**Figure 8:**
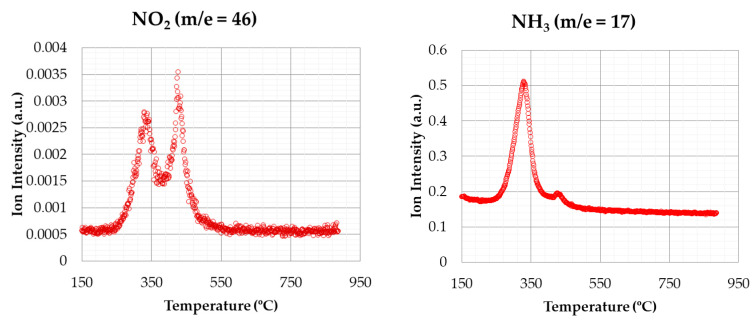
NO_2_ and NH_3_ emissions (oxidizing atmosphere).

Figure 8 shows the emissions for nitrogen containing compounds (NO_2_ and NH_3_), corresponding to the same conditions of the previous test. Concerning NO_2_ emissions, a similar behaviour to CO_2_ emissions was observed, with two emission peaks clearly pronounced, corresponding to devolatilization and char combustion stages, respectively. Regarding NH_3_ emissions, a peak was observed in the first stage of degradation, with a second peak that was much lower in the second stage. Therefore, most of the NH_3_ was evolved during the devolatilization process.

The results about CO_2_ and NO_2_ emissions in oxidizing atmosphere shown by [38] indicate that the first emission peak was more intense than the second one, opposite to the profile shown in this work.

**Figure 9:**
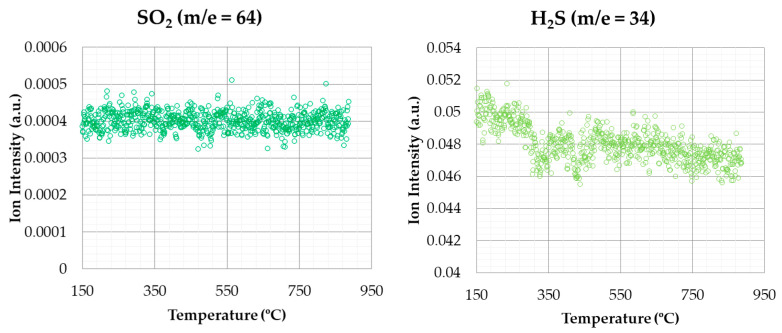
SO_2_ and H_2_S emissions (oxidizing atmosphere).

SO_2_ and H_2_S emissions, for the same conditions of the previous case, are shown in Figure 9.

With regard to sulphur-based compounds, it can be observed that there was no signal that indicates the release of SO_2_ or H_2_S emissions during the stages of thermal degradation. This fact can be due to the low percentage of sulphur in its composition (see Table 1). Nevertheless, some authors [38] found a small emission peak for SO_2_ in rice husks, probably due to the higher S percentage in this case.

The profiles obtained for CO_2_, NO_2_, and NH_3_, were similar to those obtained by other authors for other biomass wastes (for instance, spent mushroom substrate [54] or sewage sludge [57]) but not for the signal produced by SO_2_, this being almost negligible in this work.

**Figure 10:**
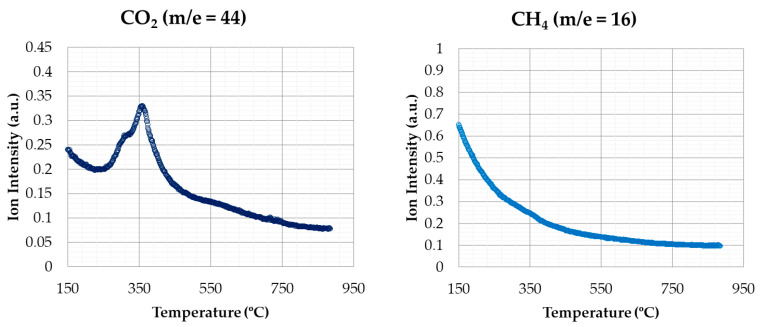
CO_2_ and CH_4_ emissions (inert atmosphere).

Figure 10 shows the emissions of CO_2_ and CH_4_ for the thermal degradation test under inert atmosphere with a heating rate of 20 °C min^−1^. As in the previous case, most CO_2_ emissions were produced during the devolatilization stage. Other authors, such as Worasuwannarak et al. [40] showed a similar behaviour concerning CO_2_ and CH_4_ emissions for this same by-product. In this case, as there was not any char degradation, the second peak was not observed for CO_2_ in oxidizing atmosphere (see Figure 7). As in the previous case, the highest values of CO_2_ emissions were generated between 350 °C, approximately. Quantitatively, a higher amount of CO_2_ was released with oxidizing atmosphere than in the case of inert atmosphere.

Other biomass samples showed the same trend, for instance in the case of olive stones [58].

**Figure 11:**
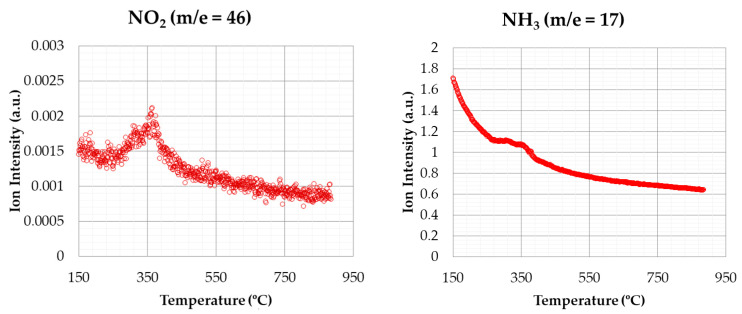
NO_2_ and NH_3_ emissions (inert atmosphere).

Figure 11 shows the emissions of nitrogen-based compounds (NO_2_ and NH_3_) for the test under inert atmosphere, undergoing the above conditions. The signal related to NO_2_ generation only showed one peak at 350 °C, approximately, which corresponded to the devolatilization stage. According to this statement, it can be assumed that all the NO_2_ generated is produced in this stage, as other variations were not observed in the remaining stages. Regarding the NH_3_ emissions, a small peak can be observed at around 350 °C. This fact suggests that there was a low NH_3_ generation during the thermal process at this temperature. This peak was also observed in the test at oxidizing atmosphere, but the signal was less pronounced. As in the case of carbon compounds, in the inert atmosphere test, the amount of the emission was less significant than at oxidizing atmosphere.

**Figure 12:**
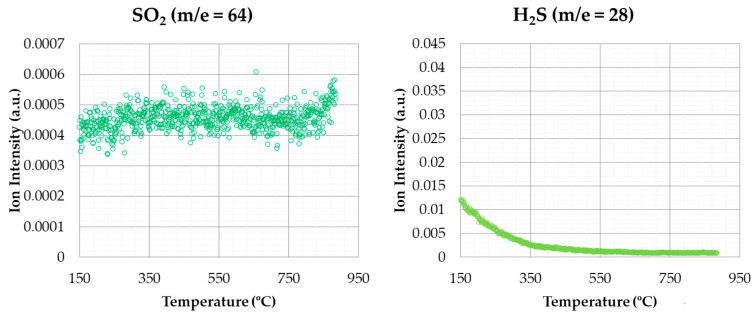
SO_2_ and H_2_S emissions (inert atmosphere).

Figure 12 shows the SO_2_ and H_2_S emissions for the thermal degradation in inert atmosphere, with the same heating rate (like the above-mentioned case). As in the tests with oxidizing atmosphere, it was not possible to observe any variation in SO_2_ and H_2_S signals. Therefore, it could be assumed that such emissions were not evolved under the atmospheres used in this study. This result, as in the case of oxidizing atmosphere, could be due to the low sulphur percentage in rice husk composition (see Table 1).

The profiles obtained for the evolved CO_2_ and NO_2_ gases under inert atmosphere were similar to those shown by Yang et al. [59] for peat, although obtaining maximum peaks at different temperatures. CO_2_ and NH_3_ profiles for chestnut shells, cherry stone, or grape seed were also similar [46]. However, other authors obtained different profiles for other kinds of biomass, such as oil palm or sawdust [60].

## 4. Conclusions

The results confirmed that rice husks can be used as a biofuel due to their suitable thermal characteristics and its low gas emission. However, its use is recommended in the same place where this by-product is generated, because of its low density. It would also be possible to use it more widely if a densification treatment were carried out (such as pelletizing) to increase its energy density. Also, the high amount of ash that was obtained should be considered, as this fact makes the use of this by-product in domestic boilers impossible. However, it is not an obstacle to be used in industrial equipments with higher power.

Rice husks showed energy activation values close to those found in similar by-products, hardly exceeding 200 kJ moL^−1^. The results showed that the average values obtained for each method (FWO, KAS, and Starink) were similar and followed the same pattern regarding the degree of conversion. Therefore, it can be assumed that these methods are valid for the calculation of thermal properties of biomass by-products such as rice husks.

Regarding the released gases in inert and oxidizing atmosphere, their levels were not problematic. The emission profiles were like those observed in other biomass by-products, with CH_4_ (for both atmospheres) and NH_3_ (for inert atmosphere) emissions being almost negligible. Finally, emissions of sulphur-based gases were not observed for any atmosphere studied.

## Figures and Tables

**Table 1:** Test carried out for characterization.

% C, H and N	UNE-EN ISO 16948 [26]
% S	UNE-EN ISO 16994 [27]
% Ash	UNE-EN ISO 18122 [28]
% Volatile Matter	UNE-EN ISO 18123 [29]
% Moisture content	UNE-EN ISO 18134-2 [30]
Heating value	UNE-EN ISO 14918 [31]
Bulk density	UNE-EN ISO 17828 [32]

**Table 2:** Characterization for Rice Husk.

**Ultimate Analysis**	**Value**
C (%) (db)	40.8 ± 0.03
H (%) (db)	5.99 ± 0.21
N (%) (db)	0.5 ± 0.02
S (%) (db)	0.064 ± 0.004
**Proximate Analysis**	**Value**
Ash (%) (db)	9.68 ± 0.11
Volatile matter (%) (db)	80.66 ± 0.58
Fixed carbon (%) (db)	9.66
Moisture (%) (wb)	9.72 ± 0.002
HHV (MJ kg^−^^1^) (db)	18.14 ± 0.04
Bulk density (kg m^−^^3^) (wb)	122.76 ± 1.48

db, dry basis; wb, wet basis.

**Table 3:** Thermal Decomposition Parameters.

Heating Rate	5	10	20	30	40
**Oxidative atmosphere**					
T_i_ (°C)	207	212	225	228	238
T_p_ (°C)	303	312	320	324	326
T_po_ (°C)	413	418	420	423	438
T_f_ (°C)	442	458	479	491	506
DTG_max_ (°C min^−1^)	5.66	13.43	26.65	36.47	47.49
**Inert atmosphere**					
T_i_ (°C)	177	185	197	209	217
T_p1_ (°C)	-	-	309	317	321
T_p2_ (°C)	327	338	348	355	357
T_f_ (°C)	372	391	399	403	408
DTG_max_ (°C min^−1^)	3.84	7.18	12.32	18.39	25.05

Where T_i_ is the initial temperature; T_p_ is the main peak temperature in oxidative atmosphere; T_po_ is the temperature of the second peak (only in oxidizing atmosphere), T_p1_ is the temperature of the first peak; T_p2_ is the temperature of the second peak (inert atmosphere); T_f_ is the final temperature of the thermal degradation; and DTG_max_ is the value of the derivative of the weight loss at T_p_ (oxidizing atmosphere) and at T_p2_ or T_p1_, in the case of 40 °C min^−1^, for the inert atmosphere.

**Table 4:** Activation Energy and *R*^2^ Values at Different Conversion Degrees.

Conversion Value	FWO Method		KAS Method		Starink Method	
*α*	*E_a_*	*R* ^2^	*E_a_*	*R* ^2^	*E_a_*	*R* ^2^
0.1	193.87	0.990	194.90	0.989	195.08	0.989
0.2	190.53	0.996	191.12	0.995	191.35	0.995
0.3	198.09	0.996	198.89	0.995	199.10	0.995
0.4	210.00	0.991	211.26	0.990	211.44	0.990
0.5	223.67	0.992	225.48	0.992	225.63	0.992
0.6	214.84	0.994	216.04	0.993	216.24	0.994
0.7	212.43	0.998	213.35	0.998	213.57	0.998
0.8	201.66	0.998	201.88	0.997	202.16	0.997
Avg.	205.64	0.994	206.61	0.994	206.82	0.994

## Data Availability

Data available on request due to restrictions eg privacy or ethical. The data presented in this study are available on request from the corresponding author.
